# Haida Gwaii (British Columbia, Canada): a Phanerozoic analogue of a subduction-unrelated Archean greenstone belt

**DOI:** 10.1038/s41598-019-39818-7

**Published:** 2019-03-01

**Authors:** J. Gregory Shellnutt, Jaroslav Dostal

**Affiliations:** 1 National Taiwan Normal University, Department of Earth Sciences, 88 Tingzhou Road Section 4, Taipei, 11677 Taiwan; 2 Saint Mary’s University, Department of Geology, 923 Robie Street, Halifax, NS B3H 3C3 Canada

## Abstract

Understanding the formation and evolution of Precambrian greenstone belts is hampered by gaps in the rock record and the uncertainty of the tectonic regime that was operating at the time. Thus identifying a modern analogue of a Precambrian greenstone belt can be problematic. In this paper we present geological, geochemical and petrological evidence outlining the case for Haida Gwaii (British Columbia, Canada) as a modern example of a greenstone belt. Haida Gwaii is comprised of two rift-related volcano-sedimentary sequences. The older (Early Triassic) Karmutsen volcanic sequence consists of subaqueous ultramafic-mafic volcanic rocks that are capped by marine carbonate and siliciclastic rocks. The younger (Paleogene) Masset bimodal volcanic sequence consists of tholeiitic and calc-alkaline basalt along with calc-alkaline silicic volcanic and intrusive rocks that are capped by epiclastic sandstones. The Karmutsen and Masset volcanic rocks have indistinguishable Sr-Nd-Pb isotopes demonstrating they were derived from a similar mantle source. Some of the Masset calc-alkaline rocks are compositionally similar to magnesian andesites (SiO_2_ = 56–64 wt%; Mg# = 0.50–0.64) that are typical of subduction-related Archean greenstone belts. We show that the calc-alkaline signature observed in the bimodal sequence of the Masset Formation is likely due to fractional crystallization of a tholeiitic parental magma under relatively oxidizing (ΔFMQ + 0.7) conditions indicating that a calc-alkaline signature is not *prima facie* evidence of a subduction setting. Given the geological and geochemical evidence, Haida Gwaii represents one of the best analogues of a modern subduction-unrelated Archean greenstone belt.

## Introduction

Beyond the volcanic nature of Archean greenstone belts their origin remains one of the most debated issues in the solid earth sciences^1–3^. It is unclear what precisely a greenstone belt is and whether all are representative of the same geological feature^4^. For example, some greenstone belts are interpreted to be ophiolites or slivers of oceanic crust whereas others are thought to be oceanic plateaux, volcanic rift complexes, or island-arcs^4–13^. In other words, some greenstone belts may be subduction-related whereas others are subduction-unrelated^14,15^.

The principal contentious issue is whether Archean greenstone belts were formed by plate tectonic (subduction, sea-floor spreading, continental drift) processes that are similar to modern Earth or if they are the consequence of convective-force (mantle plumes, diapirism, foundering) or ‘vertical’ tectonics^16–25^. In addition to their belt-like nature and accretionary-like structure, amongst the most compelling evidence in favour of a subduction setting for some greenstone belts is the presence of boninitic rocks within the mafic-ultramafic volcanic series and magnesian andesite and calc-alkaline silicic rocks in the bimodal series^26–30^. However, the apparent absence of Atlantic-style passive margin sequences, deep-water sedimentary rocks, blueschists, ultra-high pressure rocks, paired metamorphic belts, and the extent of thrust-related structures imply that the thermal and tectonic processes that were operating during the Archean were different than today^3,31–34^. Consequently, finding a modern analogue of an Archean greenstone belt is important so that it can help to address the knowledge gap in their structural, petrological and temporal evolution.

**Figure 1:**
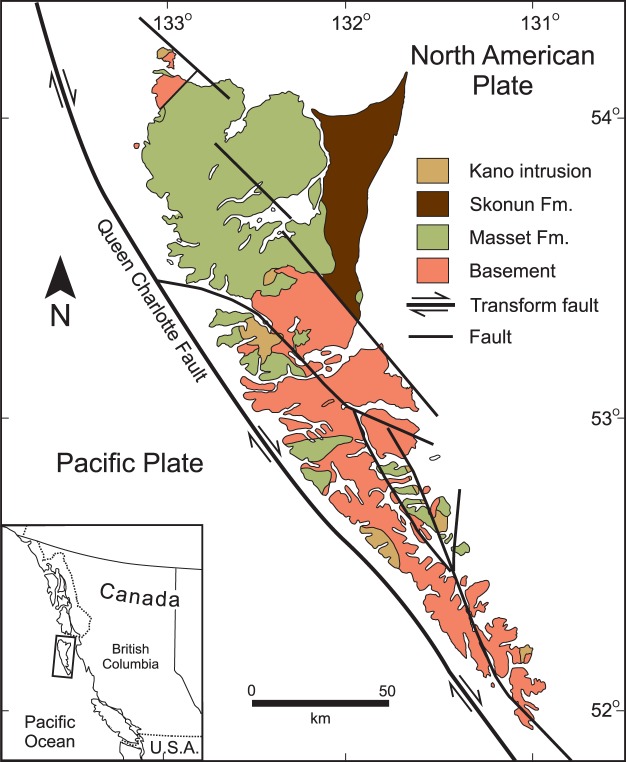
Simplified geological map of Haida Gwaii (formerly Queen Charlotte Islands)^37^. The map shows the distribution of Masset and Skonun formations, Kano intrusions and Mesozoic and older basement. A significant part of the basement is composed of Karmutsen Formation. Inset shows the location of Haida Gwaii. The Pacific plate is separated from the North American plate by the Queen Charlotte transform fault, which coincides with the continental margin and with the western side of the Queen Charlotte basin. The Masset Formation (volcanic rocks undifferentiated) and Skonun Formation (sediments) are, in part, time correlative and separate lithofacies rather than true formations. Basement, which equals to stratigraphy of the Wrangellia allochthon, includes uppermost Paleozoic limestones, Middle to Late Triassic Karmutsen basalts, Late Triassic limestones, Jurassic limestones and volcaniclastic rocks, Jurassic plutons and Early Cretaceous lithic clastic rocks. The arrows show the movement sense of the Queen Charlotte transform fault.

The islands of Haida Gwaii located ~70 km west from mainland British Columbia (Canada) are comprised of two rift-related volcanic series which are separated by and intercalated with sedimentary sequences of marine and continental origin (Fig. 1)^35–37^. The Early Triassic Karmutsen Formation basalts are part of a major large igneous province emplaced in a marine setting^38,39^. The flood basalts form a discontinuous belt from Vancouver Island and Haida Gwaii to eastern and central Alaska and SW Yukon (Nikolai Formation). On Vancouver Island, the Karmutsen Formation contains picritic basalts that have 9–20 wt% MgO^38^. The bimodal Masset Formation unconformably lies on top of the Karmutsen basalt and Mesozoic sedimentary rocks that belong to Wrangellia. Wrangellia is an allochthonous terrane that was accreted to western North America during the Late Jurassic or Early Cretaceous^40^. The composition of the volcanic rocks and the stratigraphy of the island broadly match that of an archetypical Archean greenstone belt^2,3,41^. In this paper we examine the geology, stratigraphy, structure and geochemistry of the magmatic rocks of Haida Gwaii in order to evaluate the possibility that it may be a Phanerozoic analogue of a subduction-unrelated Archean greenstone belt.

## Archean Greenstone Belts

Greenstone belts are linear to curvilinear belt-like structures that are typically 10–25 km wide, 100–300 km long, 5 km to 30 km thick, and comprised of a lower volcanic unit and an upper sedimentary unit^1–3^. Furthermore, greenstone belts are variably metamorphosed, contain precious and base metal deposits (e.g. Au, Ag, Ni, Cu), and provide a record of the processes that contributed to the formation of proto-continental crust^2,3,42–48^. Although each greenstone belt is unique, their structure, stratigraphy, and lithology are broadly similar in spite of their uncertain origin^1–4^.

**Figure 2:**
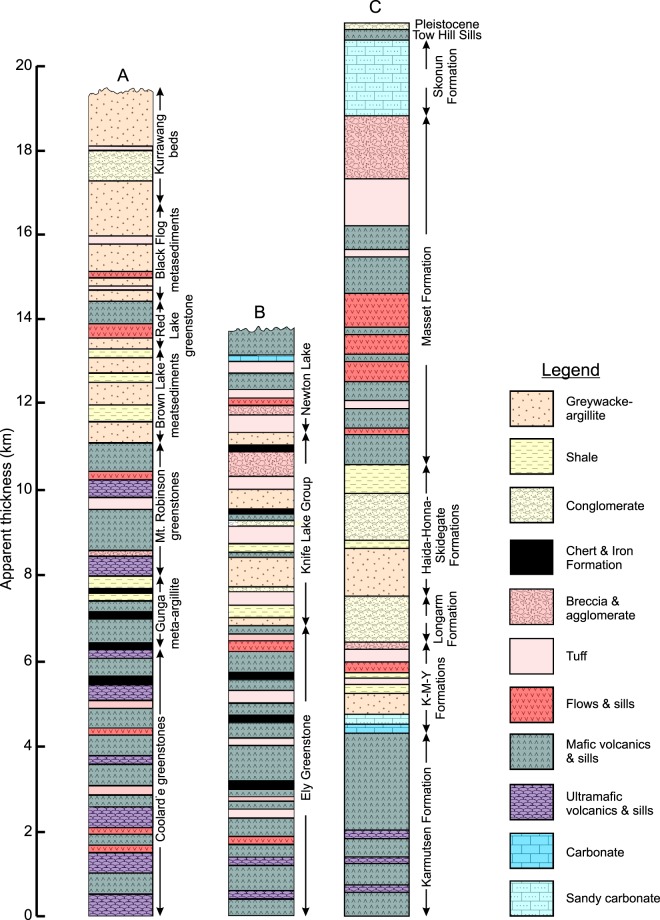
Comparison of greenstone belt stratigraphy with Haida Gwaii. (**A**) Generalized stratigraphy of the Coolgardie-Kurrawang succession of Western Australia^1^. (**B**) Generalized stratigraphy of the Vermilion succession of Minnesota^1^. (**C**) Stratigraphy of Haida Gwaii^41^.

The volcanic sequences of an archetypical^1,2^ greenstone belt are comprised of subaqueous and subaerial volcanic and volcaniclastic rocks whereas the upper unit is predominately comprised of sedimentary rocks (Fig. 2). The lower portion of the volcanic unit consists of subaqueous ultramafic (komatiite) to mafic (tholeiite, boninite) volcanic rocks with minor felsic tuffs. The upper portions of the lower unit consist of a bimodal sequence of tholeiitic flows and calc-alkaline silicic (andesite to rhyolite) volcanic rocks^1,49,50^. In some cases the mafic-ultramafic sequences are separated by thin layers of calc-alkaline rocks at time intervals ranging from 3 to 30 million years^33^. Furthermore, sedimentary depositional gaps also exist between volcanic episodes that range in duration from 2 to 27 million years^51^. The duration of magmatism of a given greenstone belt is variable but is typically <300 million years^2,3,52–54^. Many sedimentary rocks of the upper unit consist of chemically precipitated rocks such as banded iron formation, cherts and jaspers but the uppermost sedimentary rocks consist of terrigenous sediments (shales, pelitic sandstones, conglomerates, quartzites).

## Stratigraphy and Geological Structure of Haida Gwaii

The stratigraphy of Haida Gwaii matches that of the volcanic sequence of Archean greenstone belts (Fig. 2)^55,56^. The Upper Triassic Karmutsen Formation is the basal unit of Haida Gwaii and is comprised of lower subaqueous mafic tholeiitic rocks with subordinate picritic rocks and an upper subaerial mafic tholeiitic unit that is capped by limestones, calcareous siltstone, tuff and well bedded, fine grained detrital rocks^38,39,55^. Fossils constrain the age of the basalt between middle Ladinian (~230 Ma) and Carnian-Norian (~224 Ma)^57^. On Vancouver Island, the Karmutsen tholeiites are mainly submarine and 6.1–6.6 km thick. The formation consists of ca. 2900 m of basal submarine pillow lavas overlain by ~600–1100 metres of pillow breccia and aquagene tuff, followed upwards by ~2600 m of massive basalt flows and sills interbedded with shallow water and subaerial sedimentary rocks^38,58,59^. Dykes and sills are locally present however, sheeted dyke centres have not been found. The termination of volcanism was followed by submergence leading to the deposition of Late Triassic platformal carbonates.

The Karmutsen Formation is overlain by a Middle Triassic sedimentary sequence of argillite, siltstone and bivalve-bearing limestone^41^. Between the uppermost Karmutsen Formation and lowermost Masset Formation are sequences of Lower Jurassic to Upper Cretaceous conglomerates, feldspatholithic wackes, sandstones, and shales. The San Christoval and Burnaby Island plutonic suites were emplaced during the middle to late Jurassic just prior to accretion^60^. The Karmutsen plateau (Wrangellia) was accreted to the North American margin via east-ward dipping subduction^61^. During the Late Cretaceous to Early Paleogene the region became volcanically active as subaqueous volcanic debris, breccias and flows were deposited on Upper Cretaceous conglomerates, sandstones and mudstones of the Honna Formation^55,56^.

Volcanic rocks of the Early to Middle Paleogene Masset Formation occur throughout Haida Gwaii and along the western coast of British Columbia. They cover an area of ~5000 km^2^ in a continental marginal basin (half-graben) that is ~150 km wide and about 500 km long. The formation has also been intersected in offshore wells and probably^62^ covers an area of ~70 000 km^2^. In synvolcanic extensional grabens and down-faulted blocks, the volcanic sequence is up to 2 km thick but the geophysical data as well as the offshore wells indicate a thickness of ~3.5 km^63^. The volcanism is related to the development of an extensional transform pull-apart basin that experienced syntectonic Eocene extension of 2% to 15%^62,64^. The extension during Middle Tertiary led to significant crustal thinning^65^ and heating^57,66,67^. It is possible that the thermal regime during the Eocene was elevated due to the subduction of the Farallon plate ridge further to the south (~250 km from southernmost tip of Haida Gwaii) as transtensional stress opened the Queen Charlotte Basin^36,62,64^. Subduction-related mantle melting was restricted to the wedge beneath the North American margin and did not affect the lithospheric or sublithospheric mantle beneath Haida Gwaii^36^. The magmatic activities of the Masset Formation lasted from about 46.2 Ma to 11 Ma with a peak between 25 and 20 Ma^62,64,68,69^. The formation is made up of lava flows with minor pyroclastic and subvolcanic (dykes and sills) rocks. Volcanic rocks include both subaerial and subaqueous varieties. Based on the land exposure, it was estimated that the proportion of subaerial to subaqueous eruptive rocks is about 60:40^35^. The Masset rocks are distinctly bimodal with a predominance of mafic over felsic types with a ratio of about 2:1^35,37^.

There are two types of mafic rocks within the Masset Formation: aphyric to sparsely porphyritic tholeiitic basalts and strongly porphyritic calc-alkaline basaltic andesites and rare basalts^35,36^. The tholeiitic basalts contain sparse phenocrysts of olivine or olivine-plagioclase-clinopyroxene-oxides. Calc-alkaline types include phenocrysts of Ca-poor pyroxene (hypersthene), plagioclase and clinopyroxene (augite). There are also occurrences of relict aluminous amphibole with rims of orthopyroxene^35^. Silicic rocks are typically younger than the mafic types. The volcanic sequences were also intruded by plugs and stocks of approximately coeval (46-27 Ma)^60^ monzodiorites, diorites and granodiorites (SiO_2_ = 55–70 wt%)^60,68^. These intrusive rocks form two belts extending along the coast of Haida Gwaii. The Masset Formation is overlain and partially intercalated with sedimentary rocks containing both continental and marine facies.

## Petrogenesis of the Volcanic Rock Series

### Karmutsen Formation

**Figure 3:**
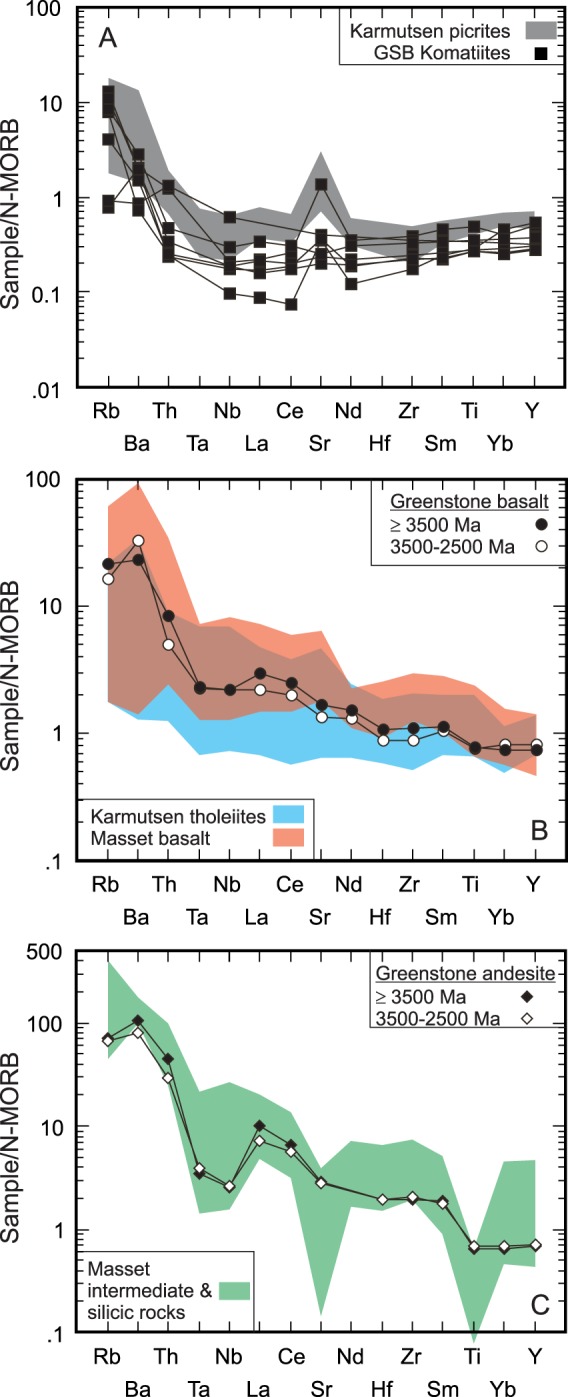
N-MORB normalized incompatible element plots of average greenstone belt (GSB) volcanic rocks, Karmutsen Formation, and Masset Formation^35–38,58,114^. (**A**) Comparison of the typical GSB komatiite^115^ to the picrites of the Karmusten Formation. (**B**) Comparison of the average tholeiitic rocks of GSB^116^ to the tholeiitic rocks of the Karmutsen and Masset Formations. (**C**) Comparison of the average andesitic and silicic rocks of GSB^116^ to the andesites and silicic rocks of the Masset Formation.

**Figure 4:**
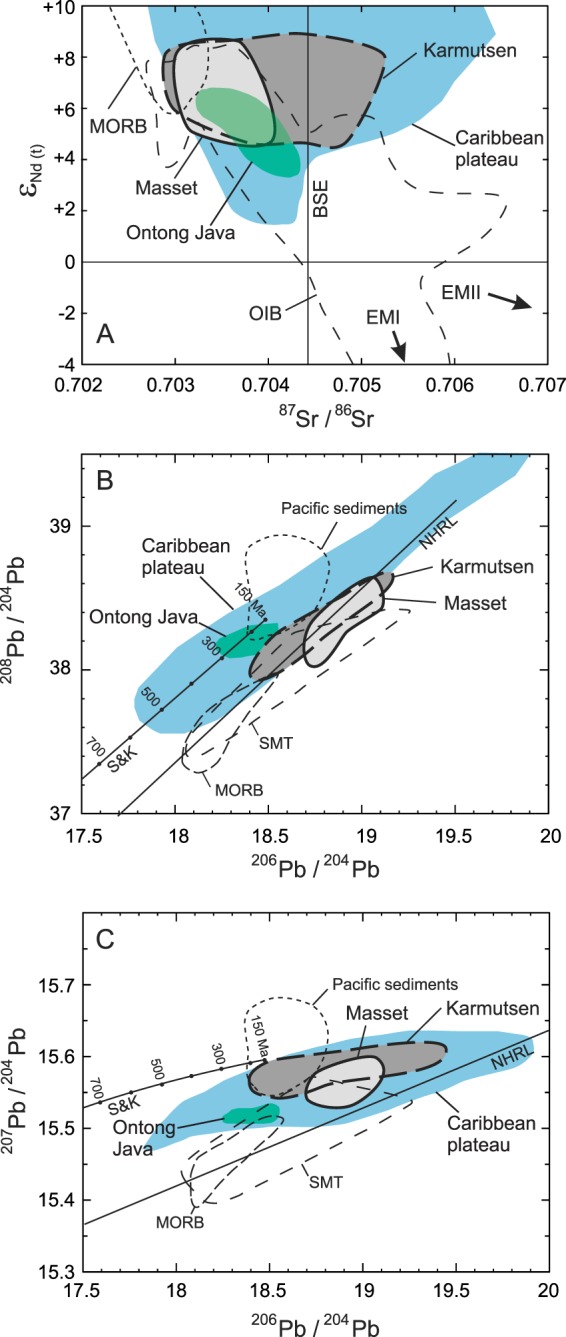
Comparison of the Sr-Nd-Pb isotopes of the Karmutsen and Masset igneous rocks. (**A**) ε_Nd_(*t*) versus initial ^87^Sr/^86^Sr ratios for volcanic rocks of the Karmutsen and Masset formations showing comparative fields for mid-ocean-ridge basalts (MORB) and ocean-island basalts (OIB). The enriched mantle EMI and EMII members as well as the Bulk Silicate Earth (BSE) evolution lines are shown for comparison. (**B**) Initial ^206^Pb/^204^Pb versus ^208^Pb/^204^Pb and (**C**) ^206^Pb/^204^Pb versus ^207^Pb/^204^Pb for the volcanic rocks of the Karmutsen and Masset formations. Comparative fields are MORB, northeast Pacific Ocean seamounts (SMT), Ontong Java plateau, Caribbean plateau, and Pacific sediments^38,117^. Data are from the Karmutsen mafic rocks whereas the Masset data include both mafic and felsic rocks^35–38,75,76^. NHRL is the Northern Hemisphere reference line^118^ while S & K denotes the reference growth curve^119^.

The Karmutsen basalts typically have SiO_2_ within a narrow range of 49–52 wt%, the Mg# (Mg^2+^/Mg^2+^ + Fe^2+^_tot_) mostly between 0.6 and 0.4, and show tholeiitic fractionation trends such as an increase of Fe, Ti and V with increasing differentiation. In addition to the basalts with 6–8 wt% MgO, the formation also contains picrites with high MgO (9 wt% to 20 wt%) contents^38,58^. The tholeiitic basalts have chondrite-normalized patterns enriched in light REE with (La/Yb)_N_ ratios ~2–2.5. On the other hand, the picrites have  (La/Yb)_N_ equal to ~0.5^38^. The shapes of mantle-normalized patterns of most tholeiitic basalts show a decrease from Nb-Ta to more compatible elements including the heavy REE (HREE) and Y. Such profiles are common among OIB and E-MORB. In contrast, the picrites have depleted-LREE patterns (Fig. 3)^38^. Both picrites and tholeiitic basalts have high positive but overlapping initial ε_Nd_(*t*) values (ε_Nd_(*t*) = +6 to +8) indicating a derivation from a common asthenospheric source^38,70^. The isotopic composition of the Karmutsen volcanic rocks is similar to a Pacific mantle-plume source (e.g. Ontong Java and Caribbean oceanic plateau) with whole rock compositions and an overall structure of an oceanic plateau (Fig. 4)^38^. The picrites were likely derived from a depleted spinel lherzolitic source by a high degree of partial melting^38,70^. In comparison, the parental magmas of the Masset Formation tholeiitic basalt are also considered to be derived from a spinel peridotite source and that their observed compositional variations are consistent with low-pressure fractional crystallization^35,36^.

The primary liquid compositions and mantle potential temperatures (*T*_P_) can be calculated from volcanic rocks providing they are representative of a liquid (i.e. not cumulate) and have only experienced olivine fractionation^71,72^. The calculated primary liquid compositions and mantle potential temperatures of the Karmutsen volcanic rocks of Haida Gwaii and Vancouver Island are summarized in Table 1. The primary liquids are picritic to komatiitic and the *T*_P_ are estimated to be between 1395 °C and 1670 °C with the higher temperatures (1570 °C to 1670 °C) indicative of an anomalously hot regime that is within the expected range of a mantle plume^71,72^.

**Table 1:** Primary melt compositions and mantle potential temperatures of the Karmutsen Formation volcanic rocks.

Sample Composition	4720A2 Thol	AFM	AFM	4721A2 Thol	AFM	AFM	5617A5(2) Sil	AFM	5614A1 Pic	AFM	AFM	93G171 Pic	AFM	AFM
SiO_2_ (wt.%)	49.24	46.54	46.97	49.56	46.07	46.49	47.4	46.08	46.24	46.36	49.48	47.93	48.58	48.68
TiO_2_	1.58	1.02	1.10	1.77	1.03	1.10	1.71	1.13	0.47	0.50	0.50	0.46	0.48	0.48
Al_2_O_3_	13.54	8.68	9.30	13.92	7.98	8.57	13.33	8.70	14.57	15.36	15.59	14.02	14.49	14.69
Fe_2_O_3_	11.30	0.50	1.08	13.07	0.50	1.08	12.71	0.55	8.57	0.25	0.50	9.93	0.24	0.48
FeO		10.95	10.39		11.57	11.04		11.23		8.05	7.80		8.82	8.61
**FeOt**														
MnO	0.21	0.21	0.21	0.17	0.16	0.17	0.17	0.16	0.15	0.16	0.16	0.19	0.19	0.19
MgO	6.09	21.55	19.68	6.06	24.38	22.62	7.46	22.95	12.11	13.50	12.96	15.84	15.19	14.68
CaO	14.00	9.05	9.68	12.15	7.02	7.54	11.93	7.84	10.81	11.40	11.56	10.42	10.76	10.91
Na_2_O	2.12	1.35	1.45	2.01	1.14	1.23	1.80	1.17	1.30	1.37	1.39	1.02	1.05	1.07
K_2_O	0.10	0.06	0.07	0.09	0.05	0.06	0.17	0.11	0.01	0.01	0.01	0.11	0.11	0.11
P_2_O_5_	0.12	0.08	0.08	0.16	0.09	0.10	0.14	0.09	0.04	0.04	0.04	0.08	0.08	0.08
Pressure (bars)		1	1		1	1		1		1	1		1	1
FeO (source)		8.02	8.18		8.02	8.02		8.02		8.27	8.25		8.35	8.35
MgO (source)		38.12	38.12		38.12	38.12		38.12		38.12	38.12		38.12	38.12
Fe_2_O_3_/TiO_2_		0.5	1.0		0.5	1.0		0.5		0.5	1.0		0.5	1.0
% ol addition		47.4	40.3		59.0	51.6		48.1		1.5	0.0		-2.3	-3.8
Melt Fraction		0.297	0.302		0.386	0.352		0.340		0.236	0.229		0.263	0.259
Temperature (^o^C)		1460	1431		1506	1481		1483		1300	1290		1335	1326
*T*_P_ (^o^C)		1602	1555		1672	1629		1637		1395	1381		1440	1426

FeOt = Fe_2_O_3_t * 0.8998 or FeOt = FeO + (0.8998*Fe_2_O_3_). AFM = accumulated fractional melting composition. The model compositions are normalized to 100% for the PRIMELT3 calculation^38,58^. Thol = tholeiite, Sil = sill, Pic – picrite.

### Masset Formation

The volcanic rocks of the Masset Formation are essentially bimodal. The mafic rocks are basalt and basaltic andesites with SiO_2_ varying between 48 and 57 wt% whereas the felsic rocks have SiO_2_ > 60 wt% (LOI-free). The intermediate rocks are volumetrically minor in comparison to the basaltic and rhyolitic rocks. The mafic types include two suites that differ not only in petrography but also in chemical compositions^35,36^. They are (1) within-plate tholeiitic basalts and (2) calc-alkaline basaltic andesite. The tholeiitic suite include aphyric basalts that range from primitive to evolved with the Mg# values spanning from 0.7 to 0.4 whereas the calc-alkaline rocks are porphyritic and typically basaltic andesite (SiO_2_ = 53.5 to 56.5 wt%). Relative to the tholeiites, the calc-alkaline rocks have lower abundances of Fe, Ti and Cr but have higher Al contents. The calc-alkaline rocks are more evolved with Mg# mostly ~0.4 and their composition is similar to that of orogenic calc-alkaline sequences. Some of the intermediate rocks are chemically similar to the magnesian andesites (SiO_2_ = 56 to 64 wt%; Mg# = 0.50 to 0.64; Ni = 50 to 100 ppm) described from the Archean Wawa greenstone belt of the Superior Craton^28,37,73^. Moreover, there is one sample that resembles the boninitic rocks (MgO > 7 wt%; Mg# = 0.62) from the Isua greenstone belt of West Greenland^29,73^. The identification of magnesian andesites and boninitic rocks is used to interpret a subduction zone setting for the Wawa and Isua greenstone belts but in the case of Haida Gwaii the rocks are definitively rift-related as their eruption was contemporaneous with the opening of the Queen Charlotte basin^62–67^.

The N-MORB-normalized trace element patterns of the intermediate rocks are broadly similar to the tholeiitic rocks but there is less variability in the Rb, Ba, and Th concentration but more variability with respect to Nb-Ta, Sr and Ti (Fig. 3)^35^. The patterns of the tholeiitic rocks resemble those of E-MORB, OIB, and many ‘high-Ti’ tholeiites of continental flood basalt provinces. Radiogenic Sr-Nd-Pb isotopes of the mafic rocks plot along the mantle array between the fields for MORB and OIB (Fig. 4). The tholeiitic and calc-alkaline mafic suites are isotopically indistinguishable. It is inferred that both suites were derived from similar parental tholeiitic magmas by polybaric fractional crystallization of different proportions of plagioclase, mafic minerals, and Fe-Ti oxide minerals^35,36^.

The highly silicic rocks are plagioclase-phyric, two pyroxene-bearing, mainly peraluminous types. They have SiO_2_ ranging from 65 to 77 wt% (LOI-free basis). The N-MORB-normalized patterns show light REE enrichment with flat heavy patterns with depletions of Ti, and Sr, and variable Nb-Ta (Fig. 3). Furthermore, the normalized patterns of the felsic rocks are enriched in large ion lithophile elements. As expected, these rocks are more enriched in highly and moderately incompatible elements than the basalts. In contrast to the basalts, most rhyolites show depletion in Ti and Sr, which suggests fractionation of plagioclase and Fe-Ti oxide minerals (ilmenite and Ti-magnetite). The felsic rocks, unlike the mafic rocks, display variable to negative Nb-Ta normalized anomalies. The variability of Nb-Ta could be related to the effects of crustal contamination or influence from a subduction modified mantle source but it is more likely that ilmenite fractionation played the primary role. The partition coefficients (D) of Nb and Ta for ilmenite in intermediate liquids is quite high (D_Nb_ = 2.3–4.6; D_Ta_ = 2.7–6.6)^74^. Consequently, as magma becomes more silicic both Nb and Ta will be depleted at a faster rate thereby generating negative normalized anomalies. Moreover, the felsic rocks have Sr-Nd-Pb isotopic compositions overlapping those of basalts including high positive ε_Nd_(*t*) values (≥+ 6). In addition, the ε_Nd_(*t*) values of the Masset Formation are similar to the Triassic Karmutsen tholeiitic basalts^38,70,75,76^. It is suggested that the mafic rocks of the Masset Formation were generated by melting of a spinel peridotite asthenospheric mantle source^36^.

### The Nature of the Calc-alkaline Signature

Calc-alkaline rocks are a subgroup of the subalkaline series that are defined by the relations between the alkali metals (Na_2_O + K_2_O), lime (CaO), the timing of Fe-Ti oxide crystallization, and water content of the magma system^77–82^. Although the term is widely used, there remain inconsistencies in its application. The calc-alkaline ‘signature’ is produced primarily due to water concentration, which affects the timing of plagioclase crystallization relative to ferromagnesian minerals, and relative oxidation state (timing of Fe-Ti oxide crystallization) but has little to do with CaO and alkalis^82^. Consequently, calc-alkaline rocks are commonly associated with volcanic-arc settings probably due to their greater likelihood of being water saturated coupled with auto-oxidation during degassing and crystallization in the crust^79,83,84^. However, calc-alkaline rocks are not restricted to arc settings and are also found at within-plate settings^81^.

**Figure 5:**
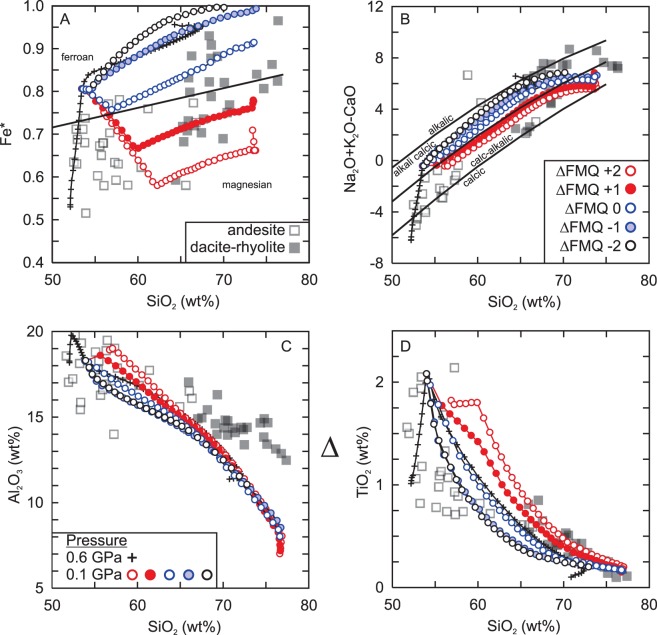
Rhyolite-MELTS polybaric fractional crystallization models of the Masset Formation tholeiitic basalt with variable relative oxidation state (ΔFMQ ± 2). (**A**) Ferroan/Magnesian (Fe* = FeOt/MgO + FeOt) division of silicic rocks^37,80^. (**B**) Modified alkali-lime index (Na_2_O + K_2_O-CaO) classification^80^. (**C**) Al_2_O_3_ (wt%) vs. SiO_2_ (wt%) and (**D**) TiO_2_ (wt%) vs. SiO_2_ (wt%). Starting compositions and conditions listed in Table 2.

The calc-alkaline silicic rocks in the Masset Formation are likely derived by fractional crystallization of a tholeiitic parental magma under relatively oxidizing conditions^37^. Here we show models to demonstrate the effects of relative oxidation state (ΔFMQ −2 to +2) on the chemical evolution of the system using Rhyolite-MELTS (Table 2). Since the parental magma is not a primary liquid we assume a three-stage fractionation process with a starting water content of 0.75 wt%^85–87^. The first stage of fractionation that produced the model starting composition likely occurred in the uppermost mantle or lowermost crust and was probably dominated by olivine but may have included pyroxene and Cr-spinel^37^. The model begins at the second stage of fractionation (0.6 GPa) with an initial redox state at the FMQ buffer. The third stage of fractionation occurs at 0.1 GPa (~3.7 km) using the pre-spinel (magnetite) liquid from the 0.6 GPa model (at 1110 °C). The ‘pre-spinel’ liquid is used because the relative oxidation state does not affect the silicate mineral compositions of the crystallizing assemblage or residual liquid composition. The results show that liquids resembling the Masset silicic rocks can be generated at higher relative oxidation states (ΔFMQ > 0) as they yield magnesian, alkali-calcic to calc-alkalic compositions that are typical of volcanic-arc settings in spite of the fact that the volcanic suite formed at a rift-setting (Fig. 5)^35–37,80^. Furthermore, the shallow pressure (0.1 GPa) models show that ilmenite fractionates relatively early (1030 °C-1080 °C) in both the oxidizing and reducing models which encompasses a silica range of ~53.5 wt% to ~59.5 wt%.

**Table 2:** Compositions and initial conditions for fractional crystallization modeling.

Sample	746^35^	1300 °C	1110 °C	421^49^	1300 °C	1110 °C
SiO_2_ (wt%)	50.74	51.90	53.49	50.2	49.46	49.47
TiO_2_	0.98	1.00	2.06	0.71	0.70	1.69
Al_2_O_3_	17.68	18.09	18.13	15.8	15.57	16.59
Fe_2_O_3_t	8.00	8.18		12.44	12.26	
FeO			7.97			14.33
Fe_2_O_3_			1.53			2.4
MnO	0.18	0.18	0.42	0.20	0.20	0.56
MgO	6.36	6.51	2.24	8.34	8.22	2.41
CaO	9.57	9.79	6.14	10.7	10.54	6.26
Na_2_O	3.20	3.27	4.95	2.24	2.21	3.92
K_2_O	0.32	0.33	0.74	0.32	0.32	0.85
P_2_O_5_	0.24	0.25	0.56	0.04	0.04	0.11
H_2_O		0.75	1.76		0.5	1.4
ƒO_2_		FMQ 0	FMQ 0 ± 2		FMQ 0	FMQ 0 ± 2
Pressure (GPa)		0.6	0.1		0.5	0.1

Major element data for models normalized to 100% including water. The starting composition at high pressure and 1300 °C is fractionated until 1110 °C. The composition of the melt at 1110 °C in the high pressure model is then fractionated at 0.1 GPa. FMQ = fayalite-magnetite-quartz buffer.

**Figure 6:**
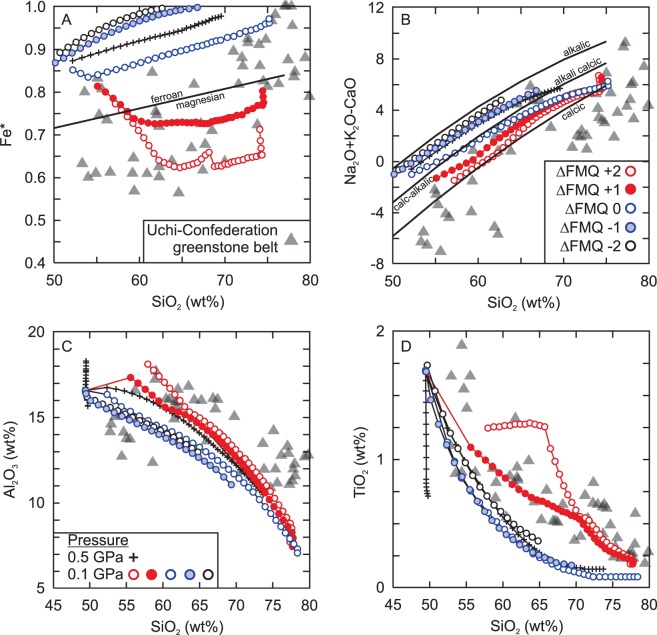
Rhyolite-MELTS polybaric fractional crystallization models of the Uchi-Confederation greenstone belt with variable relative oxidation state (ΔFMQ ± 2). (**A**) Ferroan/Magnesian (Fe* = FeOt/MgO + FeOt) division of silicic rocks^49,80^. (**B**) Modified alkali-lime index (Na_2_O + K_2_O-CaO) classification^80^. (**C**) Al_2_O_3_ (wt%) vs. SiO_2_ (wt%) and (**D**) TiO_2_ (wt%) vs. SiO_2_ (wt%). Starting compositions and conditions listed in Table 2.

Amongst other geochemical criteria (trace element ratios, isotopes), the calc-alkaline signature of some silicic greenstone belt rocks is considered to be evidence in favour of a convergent margin setting for the bimodal volcanic suite^20,88–93^. In order to test if the calc-alkaline silicic rocks from a greenstone belt can be derived by fractional crystallization, we apply the same modeling approach (H_2_O = 0.5 wt%, P_initial_ = 0.5 GPa, ΔFMQ ± 2) as the Masset Formation to the tholeiitic sequence of the Archean Uchi-Confederation (Superior Craton) greenstone belt (Table 2). The results, similar to the Masset Formation, show that the range of silicic rock compositions is reproduced under relative oxidizing (ΔFMQ > 0) conditions rather than reducing with ilmenite crystallizing only in the oxidizing (ΔFMQ +1, +2) models (Fig. 6). The implication is that the calc-alkaline signature of intermediate to silicic rocks from the Uchi-Confederation greenstone belt is likely related to the relative oxidation state of the magma system^81,82^. Although this does not preclude the possibility of a volcanic-arc setting for some bimodal sequences of greenstone belts, the models indicate that the presence of calc-alkaline rocks is not *prima facie* evidence of an arc setting. Moreover, it is possible that the depletion of Nb-Ta in some greenstone belt silicic rocks may be related to partitioning into ilmenite rather than as consequence of contamination by an ‘enriched’ source. Therefore, in order to fully utilize trace elements to constrain the tectonic setting of the intermediate to silicic volcanic rocks from a given greenstone belt, one must consider the influence that magmatic conditions (i.e., water content, relative oxidation state) impart on the melt during its evolution.

## Epithermal Gold Deposits of Haida Gwaii

Archean greenstone belts are major sources of gold^94,95^. For example ~80% of gold from Canada is mined from Archean greenstone belts of the Slave and Superior Provinces^96^. The types of gold deposits in greenstone belts are varied but typically fall under the terms ‘orogenic’ or ‘lode’ gold deposits^94,97,98^. Gold mineralization in greenstone belts is contextualized in terms of the relationship between the genesis of ore-bearing fluids and terrane accretion, thrusting, crustal shortening, metamorphism, and syn-orogenic extension. The style of gold mineralization on Haida Gwaii bears some resemblance to that of the Abitibi greenstone belt with respect to the influence of normal and listric faults as ‘mineralization corridors’ and to the emplacement of intermediate intrusions during extension^99^. However, Haida Gwaii has not experienced significant orogenic cycles or accretionary tectonics which could be a reason why there are no major gold deposits.

There are a number of different types (skarn, epithermal Au, coal, perlite, alluvial sand) of mineral deposits identified on Haida Gwaii^100^. Iron, Cu and garnet skarns within the Kunga Formation limestone and the Karmutsen volcanics represent the largest (~34%) portion of deposits but there is an appreciable amount of epithermal gold deposits (38 known deposits). The gold deposits are often structurally associated with faults and/or Paleogene intrusive rocks (Kano intrusions). Higher grade mineralization is hosted by quartz veins and veinlets but there are prospects associated with hot springs as well.

The epithermal vein deposits are mostly located on Moresby Island (southern Haida Gwaii) and primarily hosted by Yakoun Group rocks and Karmutsen volcanic rocks with one occurrence within fault breccia of Masset and Karmutsen volcanic rocks. The formation of the epithermal deposits is associated with the emplacement of the Masset Formation intrusive rocks. The plutonic rocks were emplaced within older factures and faults that probably permitted remobilization and concentration of Au and Ag in silicic fluids that formed dense networks of veins during melt migration. Furthermore, the silicic magmas likely acted as the heat source for the hot spring systems.

The hot spring associated deposits are hosted by chalcedony and fine grained quartz veins and have Au-Ag ratio of 1:10. Many of the hot spring deposits exploited pre-existing faults or fractures and likely remobilized gold as they are often structurally above the older epithermal systems and hosted by Masset Formation and Skonun Formation. In some instances the sedimentary and volcanic rocks of the Middle Jurassic Yakoun Group also host deposits as well as the limestone and sedimentary rocks of the Kunga Group.

## A Modern Analogue of an Archean Greenstone Belt

Many modern examples of greenstone belts are found along the convergent margins of the circum-Pacific region (Western Pacific and Cordilleran belts). This is, in part, due to their similarities to ophiolites but also due to the presence of boninites, calc-alkaline silicic rocks, and magnesian andesites in the arcs and accretionary complexes of Japan, Philippines and the islands of the Izu-Bonin-Mariana trench system^43,101–105^. Moreover, there are a significant number of large oceanic plateaux in the circum-Pacific region that are thought to be analogous to the lower volcanic series of greenstone belts due to their similarities in the stratigraphy, presence of subaqueous mafic-ultramafic volcanic rocks, possible association with mantle plumes, and absence of calc-alkaline rocks^8,9,106–108^. The greater abundance of oceanic plateaux in the Pacific Ocean implies there is an increased likelihood that they could act as substrate for arc initiation or act as the nucleation site of the bimodal volcanic series^109–112^. However, given the uncertainty of Archean tectonics with respect to plate tectonics, and the geological complexity of greenstone belts in general, finding a modern analogue is problematic as it is likely there are a number of different ‘types’ of greenstone belts that were formed at markedly different settings^2,4,15,16,31,32,34^. At the moment, most modern examples of greenstone belts are associated with subduction zones and/or oceanic plateaux.

The rock units and stratigraphy of the Masset Formation along with the older volcanic basement sequence of the Karmutsen Formation bear a strong resemblance to the structure and lithologies of the volcanic portion of an Archean greenstone belt. The Karmutsen Formation is similar to the ‘oceanic-plateau-like’ basement of the ultramafic-mafic series of greenstone belts whereas the Masset Formation is similar to the bimodal volcanic series (Figs 3, 5 and 6). In the case of Haida Gwaii, both volcanic series were produced under tensional stress at a rift setting which may also be true for some greenstone belts. The magmatic rocks evolve from more primitive ultramafic compositions to evolved mafic and silicic compositions over a period of ~200 million years which is within the range (~50 Ma to 300 Ma) of many Archean greenstone belts^53,54^. The chemical evolution in the upper series is mostly controlled by crystal fractionation (Fig. 5). The calc-alkaline signature of the Masset Formation silicic rocks is due to crystallization under relatively oxidizing conditions (ΔFMQ + 0.7). Moreover, the calc-alkaline signature of the silicic rocks from the Uchi-Confederation greenstone belt may be due to the relative oxidation state of the magma during crystallization (Fig. 6). Thus, the presence of calc-alkaline rocks is not in and of itself definitive evidence for a subduction-related tectonic setting for Archean or younger greenstone belts. Finally, the presence of epithermal gold deposits within all volcanic units of Haida Gwaii is reminiscent of extension-related mineralization within the Abitibi greenstone belt^99^. Therefore, we propose that Haida Gwaii is an exemplar Phanerozoic analogue of subduction-unrelated Archean greenstone belts.

## Methods

### Mantle potential temperature calculations

The primary melt compositions and mantle potential temperature estimates were calculated using PRIMELT3^72^. The major elemental data of each sample was entered into PRIMELT3 and calculated using an Fe_2_O_3_/TiO_2_ ratio of 0.5 and 1.0, pressure of 1 bar, H_2_O = 0 wt% and the lowest possible FeO content. The rock compositions and accumulated fractional melting (AFM) results are reported in Table 1.

### Rhyolite-MELTS modeling

Fractional crystallization was modeled using Rhyolite-MELTS version 1.0.1^113^. Rhyolite-MELTS is optimization for silicic magma evolution. The software permits the user to vary magmatic parameters such as relative oxidation state (ƒO_2_), pressure (GPa) and water (wt%) content so that multiple hypotheses can be tested. The starting compositions, initial magmatic conditions, and third stage compositions are reported in Table 1. We assumed initial water content of 0.75 wt% for the Masset models and 0.5 wt% for the Uchi-Confederation models and varied the relative oxidation state from ΔFMQ −2 to ΔFMQ +2. In order to simulate polybaric fractional crystallization the parental magmas are initially fractionated at high pressure (Masset = 0.6 GPa; Uchi-Confederation = 0.5 GPa) until 1110 °C. The 1110 °C composition was selected because it was the last liquid before Fe-Ti crystallization in both models. The volume of liquid remaining for the 1110 °C composition in the Masset models is 42.5% and 35.6% in the Uchi-Confederation models. The liquid composition at 1110 °C is then fractionated at a pressure 0.1 GPa (~3.7 km depth) until the model runs to completion (Stage-3). The output data files of each model are reported in the online supplementary material (Datasets S1 and S2).

## Supplementary information

Dataset S1

Dataset S2
